# Comparison of eight 15-lipoxygenase (LO) inhibitors on the biosynthesis of 15-LO metabolites by human neutrophils and eosinophils

**DOI:** 10.1371/journal.pone.0202424

**Published:** 2018-08-17

**Authors:** Anne-Sophie Archambault, Caroline Turcotte, Cyril Martin, Véronique Provost, Marie-Chantal Larose, Catherine Laprise, Jamila Chakir, Élyse Bissonnette, Michel Laviolette, Ynuk Bossé, Nicolas Flamand

**Affiliations:** 1 Centre de recherche de l’Institut universitaire de cardiologie et de pneumologie de Québec, Département de médecine, Faculté de médecine, Université Laval, Québec City, QC, Canada; 2 Centre intégré universitaire de santé et services sociaux du Saguenay–Lac-Saint-Jean, Département de sciences fondamentales, Université du Québec à Chicoutimi, Saguenay, QC, Canada; Southern Illinois University School of Medicine, UNITED STATES

## Abstract

Neutrophils and eosinophils are important sources of bioactive lipids from the 5- and the 15-lipoxygenase (LO) pathways. Herein, we compared the effectiveness of humans eosinophils and eosinophil-depleted neutrophils to synthesize 15-LO metabolites using a cocktail of different 15-LO substrates as well as their sensitivities to eight documented 15-lipoxygenase inhibitors. The treatment of neutrophils and eosinophils with linoleic acid, dihomo-γ-linolenic acid, arachidonic acid, eicosapentaenoic acid, docosahexaenoic acid and arachidonyl-ethanolamide, led to the synthesis of 13-HODE, 15-HETrE, 15-HETE, 15-HEPE, 14-HDHA/17-HDHA, and 15-hydroxy-AEA. Neutrophils and eosinophils also metabolized the endocannabinoid 2-arachidonoyl-glycerol into 15-HETE-glycerol, although this required 2-arachidonoyl-glycerol hydrolysis inhibition. Neutrophils and eosinophils differed in regard to dihomo-γ-linolenic acid and linoleic acid utilization with 15-HETrE/13-HODE ratios of 0.014 ± 0.0008 and 0.474 ± 0.114 for neutrophils and eosinophils respectively. 15-LO metabolite synthesis by neutrophils and eosinophils also differed in regard to their relative production of 17-HDHA and 14-HDHA.The synthesis of 15-LO metabolites by neutrophils was concentration-dependent and rapid, reaching a plateau after one minute. While investigating the biosynthetic routes involved, we found that eosinophil-depleted neutrophils express the 15-lipoxygenase-2 but not the 15-LO-1, in contrast to eosinophils which express the 15-LO-1 but not the 15-LO-2. Moreover, 15-LO metabolite synthesis by neutrophils was not inhibited by the 15-LO-1 inhibitors BLX769, BLX3887, and ML351. However, 15-LO product synthesis was partially inhibited by 100 μM NDGA. Altogether, our data indicate that the best 15-LO-1 inhibitors in eosinophils are BLX3887, BLX769, NDGA and ML351 and that the synthesis of 15-LO metabolites by neutrophils does not involve the 15-LO-1 nor the phosphorylation of 5-LO on Ser-663 but is rather the consequence of 15-LO-2 or another unidentified 15-LO.

## Introduction

Neutrophils and eosinophils are key effectors of several inflammatory responses. They rapidly migrate from the blood into the tissues, where they exert their multiple functions. While they are important players during host defense by promoting the clearance of microbes and parasites, they can be deleterious during chronic inflammation as their sustained activation results in tissue damage. Thus, comprehending the molecular pathways involved in neutrophil and eosinophil functions is crucial to better understand how to promote host defense while dampening, hopefully resolving inflammation.

Neutrophils and eosinophils are a rich source of inflammatory effectors, notably bioactive lipids. As such, eosinophils and neutrophils are recognized to synthesize the 5-lipoxygenase (LO)-derived leukotriene (LT) C_4_ and B_4_, respectively. Eosinophils are also recognized to synthesize several 15-LO metabolites, notably 15-hydroxy-eicosatetraenoate (HETE) and eoxin C_4_ [1, 2]. Neutrophils were also shown to synthesize 15-LO metabolites derived from arachidonic acid (AA; [3, 4]), dihomo-γ-linoleic acid (DGLA; [5]), linoleic acid (LA; [6]) and the endocannabinoid arachidonoyl-ethanolamide (AEA; [7]) which is involved in many inflammatory processes [8, 9].

Humans have two 15-LO enzymes, namely 15-LO-1 (ALOX15A) and 15-LO-2 (ALOX15B), which exhibit substantial differences in terms of expression profiles and fatty acid preferences [10, 11]. It is well recognized that eosinophils and epithelial cells express large amounts of 15-LO-1 [12]. In contrast, the expression of 15-LO-2 by leukocytes has not been thoroughly investigated yet. In that regard, the exact role of 15-LO-1 and 15-LO-2 in neutrophils is not clearly established. Indeed, while 15-LO-1 has been detected in neutrophils at the mRNA level [13], NDGA, which inhibits 15-LO-1 [14], does not inhibit and rather increases 15-HETE synthesis by neutrophils [4]. Of note, most of the previous studies documenting the synthesis of 15-LO metabolites by neutrophils were done using cell preparations contaminated with eosinophils, which constitutively express the 15-LO-1 and produce large amounts of 15-LO metabolites [2, 15]. It thus crucial to deplete eosinophils from neutrophil suspensions before studying the 15-LO pathway in these cells, as highlighted by the dramatic decrease in 15-HETE synthesis found in eosinophil-depleted neutrophils [16].

In this study, we analyzed the ability of eosinophil-depleted neutrophils to synthesize 15-LO metabolites. We found that in addition to their ability to synthesize 15-LO metabolites from LA, DLGA, AA, and AEA [3–7], they also synthesize 15-LO metabolites from eicosapentaenoic acid (EPA), docosahexaenoic acid (DHA) and 2-arachidonoyl-glycerol (2-AG). Furthermore, we compared the effect of eight inhibitors previously documented to inhibit 15-LO-1 on activated neutrophils and eosinophils and found that none of them inhibited the synthesis of 15-LO metabolites by neutrophils. Finally, we found that neutrophils express the 15-LO-2 and traces levels of 15-LO-1, in sharp opposition to eosinophils.

## Materials and methods

### Materials

2-AG, AEA, AA, EPA, DHA, DGLA, LA, MAFP, PD146176 and all internal standards for mass spectrometry were purchased from Cayman Chemical (Ann Arbor, MI, USA). DMSO was purchased from Sigma-Aldrich (St Louis, MO). Protease inhibitor cocktail tablets and adenosine deaminase (ADA) were purchased from Roche (Laval, QC, Canada). PMA, aprotinin and leupeptin were purchased from Sigma-Aldrich (St-Louis, MO, USA). DFP was purchased from BioShop Canada (Burlington, ON, Canada). Primary antibodies for β-actin (#4970), phospho-5-LO (Ser-271) (#3748), phospho-5-LO (Ser-663) (#3749), 5-LO (#3289), as well as the HRP-linked anti-mouse IgG (#7076) and anti-rabbit IgG (#7074) antibodies were obtained from Cell Signaling Technology (Beverly, MA, USA). Primary antibodies for 15-LO-1 and 15-LO-2 were purchased from Santa Cruz Biotechnology (V17, #SC-27354) and Cayman Chemical (#10004454) respectively. The HRP-linked anti-goat IgG antibody was also purchased from Santa Cruz Biotechnology (#SC-2020). PMSF, NDGA and the ECL detection kit were purchased from EMD Millipore (Billerica, MA, USA). The magnetic bead-conjugated anti-CD16 mAb and MACS were purchased from Miltenyi Biotec (Auburn, CA). HBSS was obtained from Wisent Laboratories (St-Bruno, QC, Canada). Dextran and HPLC-grade methanol and acetonitrile were purchased from Fisher Scientific. The lymphocyte separation medium was purchased from Corning (Corning, NY, USA). BLX-3887, BLX-2477, BLX-769 and BLX-2481 were kindly provided by Dr Hans-Erik Claesson (Karolinska Institutet, Stockholm, Sweden). N247 was kindly provided by Dr Frank J Dekker (Compound 14d in [17]).

### Ethics committee approval

This work required the use of human cells from healthy volunteers and was approved by our institutional ethics committee (Comité d’éthique de la recherche de l’Institut universitaire de cardiologie et de pneumologie de Québec; approval number 21200). All the experiments were conducted with the understanding and the signed consent of each participant.

### Isolation of human alveolar macrophages, monocytes, neutrophils and eosinophils

For the isolation of human monocytes, lymphocytes, neutrophils and eosinophils, human venous blood was obtained from healthy or rhinitic volunteers and collected in tubes containing K_3_EDTA as anticoagulant. Granulocytes were isolated as described previously [18]. In brief, the blood was centrifuged and the plasma was discarded. Erythrocytes were sedimented with 3% dextran, and granulocytes were separated from PBMCs using a discontinuous gradient. The PBMC layer was harvested and monocytes and lymphocytes were separated using a monocyte enrichment kit based on anti-CD14-conjugated magnetic beads (STEMCELL Technologies), according to the manufacturer’s instructions. Residual erythrocytes were eliminated from the granulocyte pellet by hypotonic lysis with sterile water. Eosinophils were separated from neutrophils using anti-CD16-conjugated magnetic beads according to the manufacturer’s instructions. The purity of the resulting neutrophil and eosinophil suspensions were assessed by Diff Quick staining and by counting 500 cells. Examples of how we can easily discriminate between eosinophils and neutrophils can be found in Fig 4 of [19]. Unless stated otherwise, the viability of neutrophil and eosinophil suspensions was always ≥ 98%, as assessed by Trypan blue exclusion analysis of 500 cells.

Human alveolar macrophages were obtained by bronchoalveolar lavage of healthy subjects. Volunteers underwent local anaesthesia before the procedure. A total of 300 ml of saline (5 syringes of 60 ml each) was injected in a segmental bronchi of the right middle lobe. The lavages were centrifuged (350 × g, 10 minutes) to pellet cells and supernatants were discarded. Cells were washed twice and viability was assessed by trypan blue exclusion. Cytospin slides were prepared and differential cell counts were performed after staining with haematoxylin and eosin. Macrophages were enriched by adhesion in 6-well plates and removal of non-adherent cells. Viability and purity were always greater than 95%, as assessed by trypan blue exclusion and Diff quick staining, respectively.

### Cell stimulations

Neutrophil or eosinophil suspensions in HBSS containing 1.6 mM CaCl_2_ were preheated at 37°C for 10 minutes. To better mimic *in vivo* conditions, adenosine deaminase (0.3 U/ml) was added 10 minutes before the addition of the stimuli in all experiments involving neutrophils [20]. Inhibitors were added 5 minutes before the fatty acids or endocannabinoids, at the concentrations detailed in figure legends. For the analysis of 15-LO metabolites by LC-MS/MS, incubations were stopped by the addition of one volume of cold (-20°C) MeOH containing 0.01% acetic acid and 2 ng of LTB_4_-D_4_ and 15-HETE-D_8_ as internal standards. Samples were then frozen until further processing. Viability was assessed by trypan blue exclusion in every experimental condition involving inhibitors, to rule out their possible toxicity.

### Analysis of 15-LO metabolites by liquid chromatography—Tandem mass spectrometry (LC-MS/MS)

Samples were thawed, centrifuged (1000 × *g*; 5 minutes) to remove the denatured proteins and the resulting supernatants were diluted with water to a final MeOH concentration of ≤ 10% and maintained at pH 2 by the addition of acetic acid. Samples were loaded on solid phase extraction cartridges (Strata-X Polymeric Reversed Phase, 60 mg/1ml, Phenomenex). Cartridges were washed with 2 ml of water and lipids were eluted with 1 ml MeOH. The eluates were dried under a stream of nitrogen and reconstituted in 50 μl of MeOH + 0.01% acetic acid. 25 μl was injected onto an RP-HPLC column (Kinetex C8, 50 × 2.1 mm, 2.6 μm) and eluted at a flow rate of 400 μl/min with a discontinuous gradient from 10% solvent A (MeCN + 0.05% formic acid) to 75% in 20 min, from 75% to 95% in 10 seconds, and held for 5 minutes at 95% A before re-equilibration to 10% A and 90% solvent B (water + 0.05% formic acid) in 3 minutes. The HPLC system was directly interfaced into the electrospray source of a triple quadrupole mass spectrometer (Shimadzu 8050) and mass spectrometric analysis was performed in the negative ion mode using multiple reaction monitoring for the specific mass transitions of each metabolite (Table 1).

**Table 1:** Specific mass transition and limit of quantification of the metabolites analyzed by LC-MS/MS.

COMPOUND	INTERNAL STD USED	Q1 → Q3	RETENTION TIME (min)	LOQ (fmol)	LOD (fmol)
15-HETE	15-HETE-D_8_	319.20 **→** 219.20	16.976 ± 0.002	156.02	78.01
15-HETE-EA	15-HETE-D_8_	346.40 **→** 62.25	14.943 ± 0.002	68.78	13.76
15-HETrE	15-HETE-D_8_	321.20 **→** 221.20	17.492 ± 0.002	77.52	77.52
13-HODE	15-HETE-D_8_	295.20 **→** 195.10	16.654 ± 0.002	168.63	84.32
15-HEPE	15-HETE-D_8_	317.20 **→** 219.20	16.242 ± 0.002	156.99	78.49
17-HDHA	15-HETE-D_8_	321.20 **→** 245.20	17.126 ± 0.001	145.14	72.57
14-HDHA	15-HETE-D_8_	343.20 **→** 205.20	17.288 ± 0.002	145.14	72.57
15-HETE-sn-1-G	15-HETE-D_8_	417.20 **→** 23.00	15.803 ± 0.020	127.13	63.56
15-HETE-sn-2-G	15-HETE-D_8_	417.20 **→** 23.00	15.738 ± 0.022	127.13	63.56
LTB_4_	LTB_4_-D_4_	335.20 **→** 195.10	14.177 ± 0.002	74.29	14.86
LTB_4_-D_4_	-	339.20 **→** 197.10	14.139 ± 0.002	-	-
15-HETE-D_8_	-	327.20 **→** 226.20	16.897 ± 0.002	-	-

STD = Standard; LOQ = limit of quantification; LOD = limit of detection.

Quantification was performed using calibration curves, as previously described [21]. In brief, each metabolite was diluted to prepare a calibration curve and were spiked with 2 ng of the deuterated standards. The samples were then extracted as described above, and analyzed on the LC-MS/MS system three times. The slope was then calculated using the ratio between the peak areas of the metabolite and the deuterated standard (15-HETE-D_8_ for 15-LO metabolites and LTB_4_-D_4_ for LTB_4_). The data generated from these standard curves was also used to calculate the limits of quantitation (LOQs) for each product (Table 1).

### Analysis of proteins by immunoblot

Cells were lysed with 0.01% NP-40 in a hypotonic lysis buffer (10 mM Tris-HCl, pH 7.4, 10 mM NaCl, 3 mM MgCl_2_, 1 mM EDTA) containing 10 μg/ml leupeptin, 10 μg/ml aprotinin, 1 mM PMSF, 3 mM DFP, 1 tablet protease inhibitor cocktail and 1 tablet phosphatase inhibitor cocktail. Laemmli sample buffer (62.5 mM TRIS-HCl [pH 6.8], 2% SDS, 10% glycerol, 0.01% bromophenol blue) was added to cell lysates and samples were boiled for 10 minutes. Buffer volumes were adjusted to obtain a final concentration of 2 million cells/50 μl of lysate for all samples. Proteins were separated by SDS-PAGE on 12% polyacrylamide gels and transferred onto PVDF membranes. Transfer efficiency was verified by Ponceau Red staining. Membranes were placed in TBS-Tween buffer (25 mM Tris-HCl [pH 7.6], 0.2 M NaCl, 0.15% Tween 20) containing 5% non-fat dried milk (w/v) for 30 minutes at room temperature, then probed with the primary antibody (4°C, overnight). The membranes were revealed by chemiluminescence using a HRP-coupled secondary antibody and an ECL detection kit.

### Transfection of NIH/3T3 cells

NIH/3T3 cells obtained from ATCC (Manassas, VA) were grown under 5% CO_2_ in Dulbecco's modified Eagle's medium (Invitrogen) with 10% calf serum and 100 units/ml each of penicillin and streptomycin. Cells were plated at 80% confluency and transfected with the pcDNA3.1-5-LO plasmid [22] using the Polyfect transfection reagent (Qiagen) following the manufacturer's instructions. Cells were harvested 16h post-transfection and were lysed and processed for immunoblotting as described above.

### Statistical analyses

Statistical analyses were done using the GraphPad Prism 7 software. *p* values < 0.05 were considered significant.

## Results

### Neutrophils and eosinophils metabolize fatty acids and endocannabinoids into 15-LO metabolites

**Fig 1:**
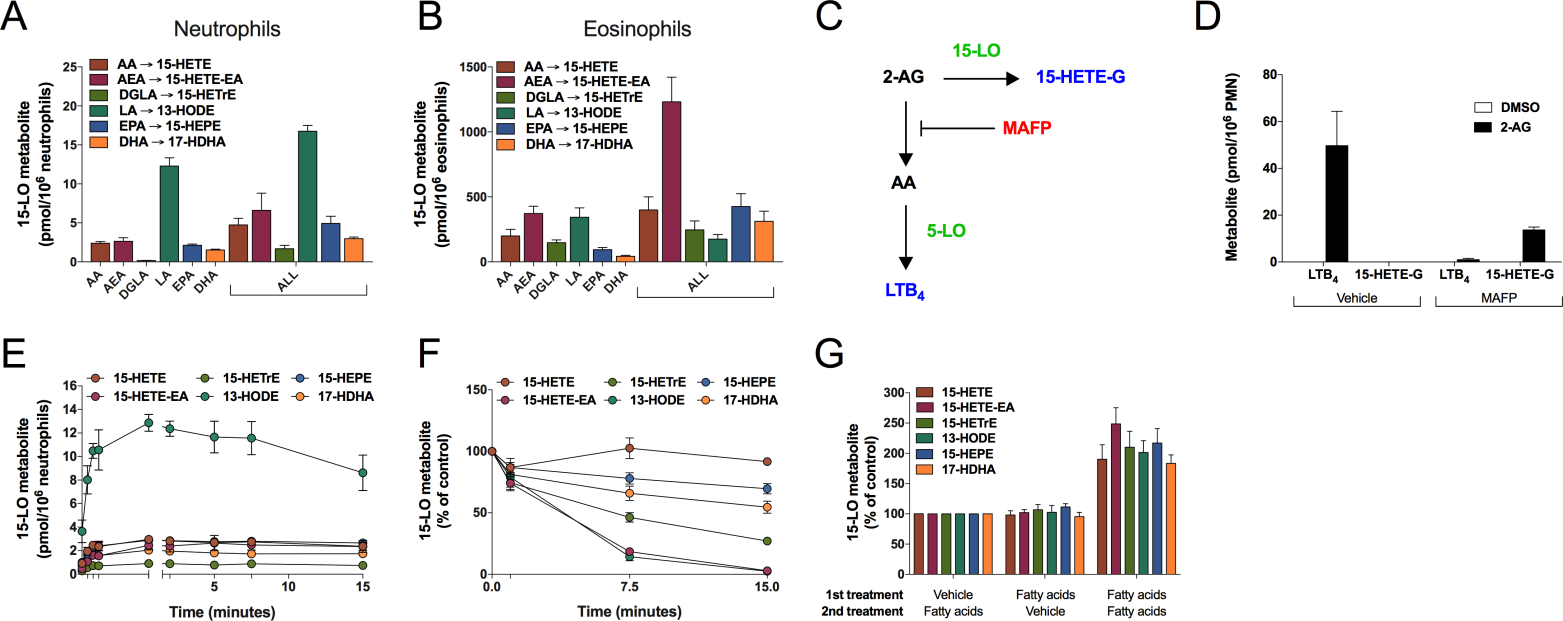
Human neutrophils and eosinophils metabolize polyunsaturated fatty acids and endocannabinoids through the 15-LO pathway. Human **(A)** neutrophils (5 × 10^6^ cells/ml) or **(B)** eosinophils (10^6^ cells/ml) were incubated with 3 μM of AEA or fatty acids, either separately or combined (ALL) during 15 minutes. **(C)** Catabolism pathways for 2-AG in freshly isolated neutrophils; **(D)** Neutrophils (5 × 10^6^/ml) were incubated with 3 μM 2-AG or its vehicle (DMSO) for 15 minutes. MAFP 1 μM or its vehicle (DMSO) were added 5 minutes before 2-AG. 15-HETE-G represents the combination of 15-HETE-1-G and 15-HETE-2-G; **(E)** Neutrophils (5 × 10^6^/ml) were incubated with a cocktail of AEA and fatty acids (LA, DGLA, AA, EPA, and DHA) at 3 μM each for the indicated times. **(F)** Neutrophils (5 × 10^6^/ml) were incubated with a cocktail of 15-LO metabolites (5 ng of each) for the indicated times. **(G)** Neutrophils (5 × 10^6^/ml) were incubated with a cocktail of fatty acids and AEA (3 μM of each) or vehicle for 1.5 minutes before the addition of the same cocktail or vehicle for another 1.5 minutes. **(A-G)** Incubations were stopped by the addition of 1 volume of a cold (-20°C) stop solution (MeOH containing 2 ng of LTB_4_-D_4_ and 15-HETE-D_8_). Samples were processed and analyzed for 15-LO metabolite and LTB_4_ production as described in *Materials and methods*. The data represents the mean (± SEM) of 3–5 separate experiments.

Neutrophils synthesize 15-LO metabolites from AA, LA, and DGLA [3–6] and no clear evidence support the involvement of either 15-LO-1 or 15-LO-2 in that phenomenon. Given the recognized ability of eosinophils to synthesize 15-LO metabolites, we sought to confirm the ability of eosinophil-depleted neutrophils to generate 15-LO metabolites of AA, LA, DGLA and AEA and to expand our investigation by assessing their ability to metabolize EPA, DHA and 2-AG. In our first series of experiments, we treated neutrophils or eosinophils from the same volunteer during 15 minutes with 3 μM of each substrate, alone or in combination. The use of a fatty acid cocktail was tested because *in vivo*, leukocytes are in presence of numerous substrates simultaneously. As shown in Fig 1, AA, LA, DGLA, EPA, DHA and AEA were converted into 15-HETE (AA), 13-HODE (LA), 15-HETrE (DGLA), 15-HEPE (EPA), 14-HDHA (DHA), 17-HDHA (DHA) and 15-HETE-EA (AEA) in both neutrophils (Fig 1A) and eosinophils (Fig 1B). The levels of 15-LO metabolites being synthesized by neutrophils were limited compared to those synthesized by eosinophils. Neutrophils and eosinophils did not metabolize DGLA and LA to the same extent, as we obtained 15-HETrE/13-HODE ratio of 0.014 ± 0.0008 for neutrophils and 0.474 ± 0.114 for eosinophils. While the endocannabinoid AEA was metabolized into 15-HETE-EA (Fig 1A and 1B), the endocannabinoid 2-AG was not converted into 15-HETE-G (Fig 1D), although it yielded significant amounts of LTB_4_ (~50 pmol/million neutrophils). This was not surprising given that we previously showed that neutrophils hydrolyze 2-AG into AA within seconds [18]. Thus, in order to assess if 2-AG was metabolized into 15-HETE-G by neutrophils, we prevented 2-AG hydrolysis into AA with the serine hydrolase inhibitor MAFP (Fig 1C and [18, 23]). As expected, 2-AG-hydrolysis inhibition with MAFP (1 μM) blocked the transformation of 2-AG into LTB_4_ and led to the synthesis of 15-HETE-G (Fig 1D). This indicates that 2-AG is also a substrate for the 15-LO pathway in neutrophils, although it only occurs in conditions in which the hydrolysis of 2-AG into AA is prevented. Essentially similar results were obtained in human eosinophils (data not shown). For these reasons, we did not include 2-AG in the remainder of this study.

Statistical analysis (2-way ANOVA with Sidak multiple comparison test) between the fatty acids alone or the fatty acid cocktail indicated that the synthesis of 15-LO metabolites were comparable, with the exception of 15-HETE-EA (p < 0.05) and 13-HODE (p < 0.01) for neutrophils, and 15-HETE-EA (p < 0.0001) and 15-HEPE (p < 0.05) for eosinophils and comparable for the other 15-LO metabolites. Unless stated otherwise, we used the fatty acid cocktail for the following experiments to increase throughput given the limited numbers of eosinophils obtained from each donor. We next conducted kinetic experiments in neutrophils using a substrate cocktail consisting of AA, LA, DGLA, EPA, DHA and AEA (Fig 1E). The synthesis of every 15-LO metabolite reached a plateau (1 minute) then remained stable for up to 15 minutes, indicating that 15-LO metabolite synthesis was either halted (enzyme inactivation) or that 15-LO metabolite synthesis was equal to 15-LO metabolite disappearance. To investigate those possibilities, we first assessed the half-life of 15-LO metabolites in neutrophils and found that most metabolites disappeared from the incubation media with a half-life of ~5 minutes (Fig 1F). We next investigated the hypothesis of enzymatic inactivation by treating neutrophils with vehicle or our fatty acid cocktail for 1.5 minutes, then re-treating neutrophils with vehicle or the fatty acid cocktail for 1.5 minutes, for a total stimulation of 3 minutes (Fig 1G). Neutrophils did synthesize additional amounts of 15-LO metabolites after the second stimulation with fatty acids, confirming that the enzyme was not inactive. This supports that the plateau we observe in our time-course assay is likely the consequence of a balance between *de novo* synthesis and degradation of the metabolites, rather than enzyme inactivation.

### Expression of 15-LO-1 and -2 by freshly isolated neutrophils and eosinophils

**Fig 2:**
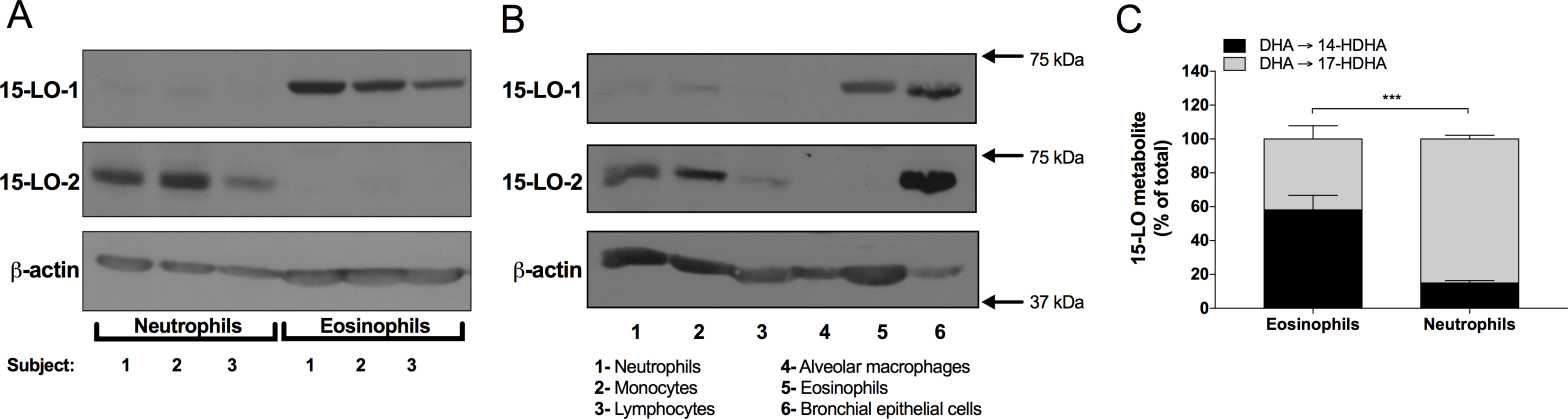
Differential expression of 15-LO-1 and -2 in human leukocytes. **(A,B)** Freshly isolated leukocytes were disrupted and analyzed by immunoblot for 15-LO-1, 15-LO-2 and β-actin as described in *Materials and methods*. The data from Panel A represent human neutrophils and eosinophils obtained from three subjects. Panel B is from one experiment that is representative of three independent experiments. **(C)** Neutrophils (5 × 10^6^/ml) were incubated with 3 μM of DHA for 15 minutes. Incubations were stopped by the addition of 1 volume of a cold (-20°C) stop solution (MeOH containing 2 ng of LTB_4_-D_4_ and 15-HETE-D_8_). Samples were processed and analyzed for 15-LO metabolite and LTB_4_ production as described in *Materials and methods*. The data represents the mean (± SEM) of 4 separate experiments. A two-way ANOVA with Sidak’s multiple comparisons test was performed to obtain *p* values. *** *p* < 0.001.

While eosinophils are known to abundantly express 15-LO-1 and to synthesize 15-LO metabolites [2, 12, 15, 24, 25] the data regarding 15-LO-1 and -2 expression by neutrophils is conflicting and might reflect, at least in part, the presence of eosinophils in neutrophil suspensions, as supported with the depletion of eosinophils from neutrophil suspensions [16]. In this regard, we analyzed the expression of both 15-LO-1 and 15-LO-2 in eosinophil-depleted neutrophils and eosinophils. Our immunoblot data show that eosinophils express 15-LO-1 but not 15-LO-2, and that neutrophils express 15-LO-2 but not 15-LO-1 (Fig 2A). We further investigated these expression profiles and compared 15-LO-1 and -2 protein levels in other cell types. We found that 15-LO-2 expression is also found in freshly isolated monocytes and lymphocytes but not in freshly isolated AMs, while IL-13-treated bronchial epithelial cells express both, 15-LO-1 and 15-LO-2 (Fig 2B). An interesting way to discriminate between 15-LO-1 and 15-LO-2 activities is to assess the ratio between 14-HDHA and 17-HDHA [26]. We thus incubated neutrophils and eosinophils in presence of DHA alone and assessed their ability to synthesize 17-HDHA and 14-HDHA. Under these experimental conditions, we found significant differences between the 17-HDHA/14-HDHA ratio in neutrophils vs eosinophils (Fig 2C), supporting the involvement of different enzymes.

### Impact of 15-LO inhibitors on 15-LO metabolite biosynthesis in neutrophils and eosinophils

**Fig 3:**
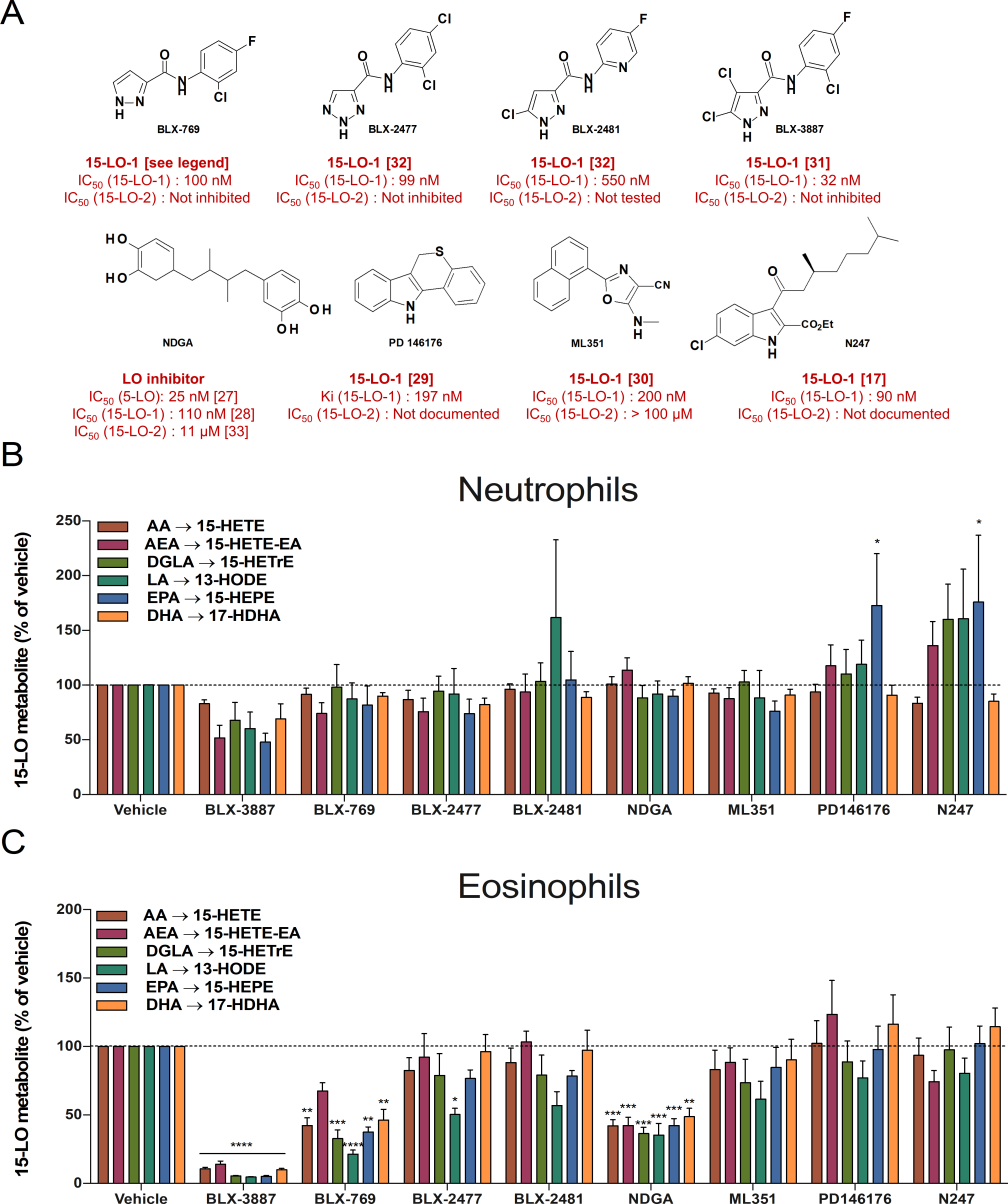
Impact of various 15-LO inhibitors on 15-LO metabolism in human neutrophils and eosinophils. **(A)** Chemical structures of the 15-LO inhibitors used in this study. IC_50_ for BLX-769 was a personal communication from Hans-Erik Claesson. **(B, C)** Human neutrophils (5 × 10^6^ cells/ml) or human eosinophils (10^6^ cells/ml) were pre-incubated with DMSO (or with 10 μM of the different 15-LO inhibitors for 5 minutes. A cocktail of fatty acids and AEA (3 μM of each) was then added to the cell suspensions for 15 minutes. Incubations were then stopped with 1 volume of a cold (-20°C) solution (MeOH containing 2 ng of LTB_4_-D_4_ and 15-HETE-D_8_). Samples were processed and analyzed by LC-MS/MS for 15-LO metabolite biosynthesis as described in *Materials and methods*. The data represents the mean (± SEM) of at least three separate experiments. A two-way ANOVA with Dunnett’s multiple comparisons test was performed to obtain *p* values. * p < 0.05; ** p < 0.01; *** p < 0.001; **** p < 0.0001 vs the corresponding 15-LO metabolite without inhibitor (vehicle).

To rule out the involvement of 15-LO-1 in 15-LO metabolite synthesis by neutrophils, we assessed the impact of several 15-LO inhibitors. Given that human eosinophils are known to synthesize abundant levels of 15-LO metabolites through 15-LO-1, we used them as a positive control for 15-LO-1 inhibition. The compounds that were available to us (Fig 3A) were: the nonselective LO inhibitor nordihydroguaiaretic acid (NDGA) [27, 28] and the 15-LO-1 inhibitors PD146176 [29], ML351 [30], N247 [17], BLX-769, BLX-2477, BLX-2481 and BLX-3887 [31, 32]. As a starting point, we tested the 15-LO inhibitors at 10 μM, to pinpoint those that had significant potencies at inhibiting 15-LO metabolite synthesis. When used at the working concentration of 10 μM, none of these inhibitors significantly blocked 15-LO metabolite synthesis by neutrophils (Fig 3B). In sharp contrast, BLX-3887 inhibited >90% of 15-LO metabolite biosynthesis in eosinophils (Fig 3C). NDGA and BLX-769 also blocked approximately 50% of the 15-LO activity in eosinophils at 10 μM, whereas the other compounds displayed minimal or no inhibitory effect. Importantly, none of the inhibitors, at 10 μM, affected the viability of eosinophils and neutrophils during a 20-minute incubation, as assessed by trypan blue exclusion (data not shown). Finally, the incubation of eosinophils and neutrophils with 10 μM NDGA led to a significant inhibition of LTC_4_ (92 ± 2%) and LTB_4_ (100%) synthesis respectively, indicating that NDGA was potent enough to abrogate 5-LO in the experiment conditions we performed.

**Fig 4:**
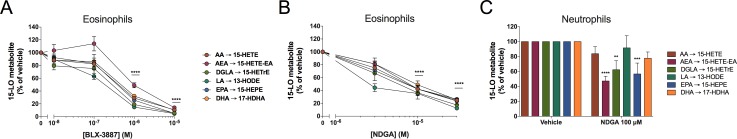
Concentration-response curves for BLX-3887 and NDGA in eosinophils and neutrophils. **(A,B)** Eosinophils (10^6^ cells/ml) or **(C)** neutrophils (5 × 10^6^ cells/ml) were pre-incubated with the indicated concentrations of **(A)** BLX-3887 or **(B,C)** NDGA for 5 minutes. A cocktail of fatty acids and AEA (3 μM of each) was then added to the cell suspensions for 15 minutes. Incubations were stopped after 15 minutes with 1 volume of a cold (-20°C) solution (MeOH containing 2 ng of LTB_4_-D_4_ and 15-HETE-D_8_). Samples were processed and analyzed by LC-MS/MS for 15-LO metabolite biosynthesis as described in *Materials and methods*. The data represents the mean (± SEM) of at least three separate experiments. A two-way ANOVA with Sidak’s multiple comparisons test was performed to obtain *p* values. ** p < 0.01; *** p < 0.001; **** p < 0.0001 vs the corresponding 15-LO metabolite without inhibitor (vehicle).

The effect of the 15-LO-1 inhibitor BLX-3887 on 15-LO metabolites synthesis in eosinophils was concentration-dependent as opposed to experiments performed in neutrophils (Fig 4A for eosinophils). Because NDGA was previously documented to partially block 15-LO-2 activity at high μM concentration in enzymatic assays [33], we tested concentrations higher than 10 μM of the latter on AEA- and fatty acid-stimulated neutrophils and eosinophils (Fig 4B and 4C). While it inhibited the biosynthesis of 15-LO metabolites in eosinophils in a concentration-dependent manner, NDGA only partially blocked the 15-LO activity of human neutrophils at 100 μM, and its efficacy varied widely among substrates. We were unable to assess higher concentrations of NDGA, as it affected cell viability at concentrations higher than 100 μM (data not shown). Altogether, these results show that at least some of the inhibitors we tested have enough potency and efficacy to block 15-LO-1 and 15-LO metabolite biosynthesis by eosinophils. In contrast, all selective 15-LO-1 inhibitors did not affect the synthesis of 15-LO metabolites by neutrophils, supporting the notion that 15-LO-1 is not involved in the biosynthesis of 15-LO metabolites by neutrophils.

### Impact of 5-LO and its phosphorylation on the biosynthesis of 15-LO metabolites by neutrophils

**Fig 5:**
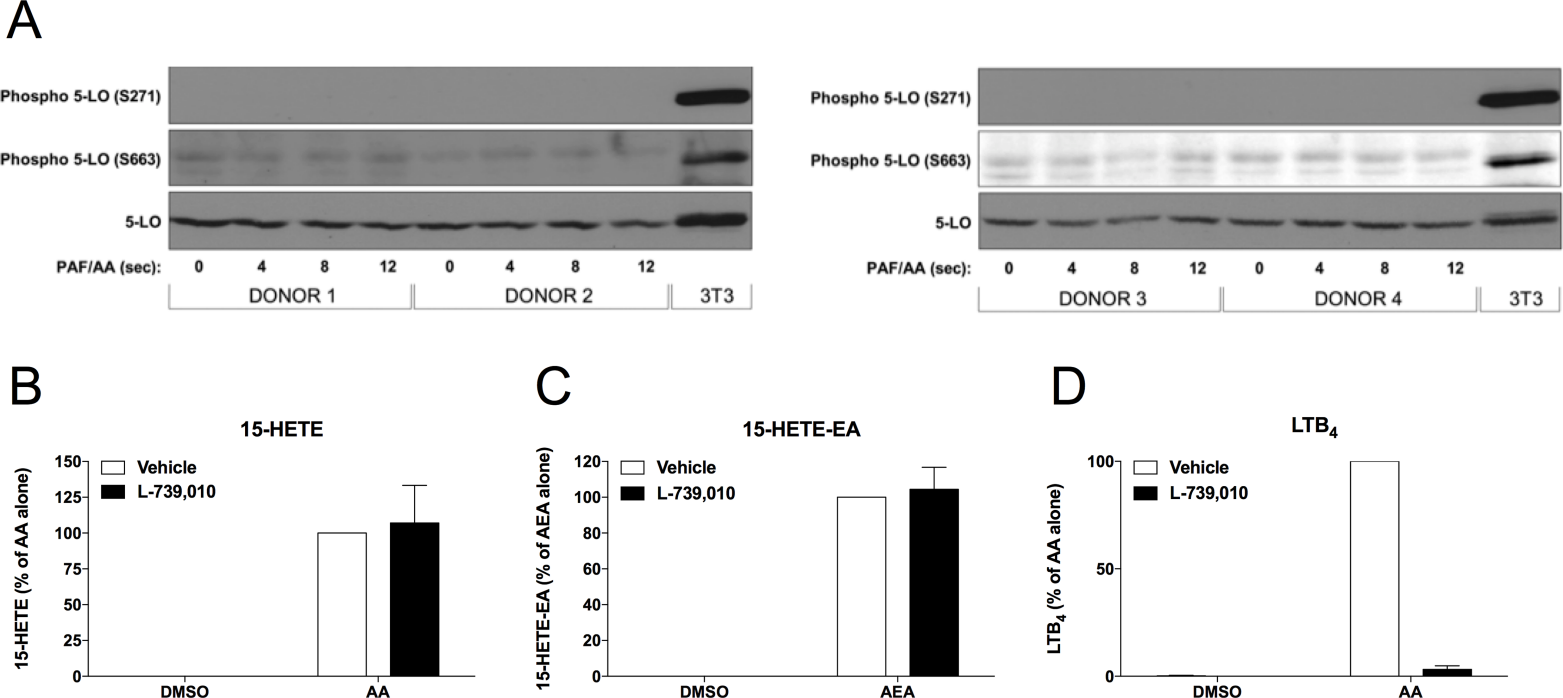
5-LO phosphorylation and activity are not involved in the synthesis of 15-LO metabolites by human neutrophils. **(A)** Neutrophils (10^7^ cells/ml) were stimulated with the combination of 10 μM AA and 1 μM PAF for the indicated times. Incubations were stopped by adding 2 volumes of cold (4°C) incubation buffer. Cells were disrupted and analyzed by immunoblot for total and phopho-5-LO as described in *Materials and methods*. **(B-D)** Neutrophils (5 × 10^6^/ml) were pre-incubated with 100 nM of the 5-LO inhibitor L-739,010 for 5 minutes then stimulated with 10 μM AA or 10 μM AEA for 15 minutes. Incubations were stopped by adding 1 volume of a cold (-20°C) solution (MeOH containing 2 ng of LTB_4_-D_4_ and 15-HETE-D_8_). Samples were then processed and analyzed by LC-MS/MS for 15-LO metabolite biosynthesis as described in *Materials and methods*. The data represent the mean (± SEM) of at least three independent experiments.

Using a Ser-663 phosphorylation mimic, it was shown that 5-LO can acquire a 15-LO activity [34]. Thus, we investigated if 5-LO phosphorylation on Ser-663 occurred in our experimental conditions and if 5-LO was involved in the biosynthesis of 15-LO metabolites by neutrophils. We thus performed a series of experiments in which neutrophils were stimulated with the combination of AA and PAF (to maximize 5-LO activity), and further assessed 5-LO phosphorylation by immunoblot. Given that 15-LO metabolism in neutrophils is rapidly reaching a plateau after the addition of the substrate (Fig 1), we assessed 5-LO phosphorylation at early time points (0–12 seconds). As shown in Fig 5A, the combination of AA and PAF failed to phosphorylate Ser-663 (and Ser-271) on 5-LO in neutrophils, suggesting that the phosphorylation of 5-LO on Ser-663 (and Ser-271) is not responsible for the 15-LO activity we observed in neutrophils. We further confirmed this by assessing the effect of the 5-LO inhibitor L-739,010 [35, 36] on 15-HETE and 15-HETE-EA biosynthesis upon the addition of AA and AEA, respectively. L-739,010 had no effect on 15-LO metabolite production by AEA- and AA-stimulated neutrophils (Fig 5B and 5C). However, it did inhibit the AA-induced LTB_4_ synthesis (Fig 5D), confirming its potency and efficacy toward 5-LO in neutrophils. Altogether, these findings exclude the possibility that 5-LO is involved in the biosynthesis of 15-LO metabolites by human neutrophils we observed.

## Discussion

Despite a growing body of evidence showing that 15-LO metabolites are involved in the regulation of neutrophil functions and neutrophilic inflammatory disorders, the involvement of neutrophils in the synthesis of 15-LO metabolites was ill defined. Given the wide array of inflammatory lipids that are synthesized via the 15-LO pathway, characterizing this metabolic pathway in neutrophils betters our understanding of their role in inflammatory diseases.

In this study, we propose a potential role for 15-LO-2 in the production of 15-LO metabolites by human neutrophils. This is based on the following observations: **1)** neutrophils metabolize endocannabinoids and fatty acids via the 15-LO pathway; **2)** neutrophils express 15-LO-2 while eosinophils express 15-LO-1; **3)** The 17-HDHA/14-HDHA ratio is clearly different between neutrophils and eosinophils; **4)** 15-LO-1 inhibitors effectively prevent 15-LO metabolite biosynthesis in eosinophils but not in neutrophils; **5)** the production of 15-LO metabolites by neutrophils is decreased by high concentrations of NDGA; and **6)** 5-LO inhibition with 15-LO-1 inhibitors did not affect the synthesis of 15-LO metabolites by neutrophils.

While neutrophils and eosinophils metabolized all substrates, we noted a striking difference between the 15-HETrE/13-HODE ratio (0.014 ± 0.0008 and for neutrophils and 0.474 ± 0.114 for eosinophils) as well as the 17-HDHA/14-HDHA ratio (5.85 ± 0.661 for neutrophils and 0.716 ± 0.052 for eosinophils). When incubating recombinant 15-LO enzymes with DHA, Kutzner et all found a biosynthetic 17-HDHA/14-HDHA ratio of 1.5 and 48.3 for 15-LO-1 and 15-LO-2, respectively [26]. While these ratios cannot confirm the involvement of 15-LO-1 and 15-LO-2 in eosinophils and neutrophils, respectively, they strongly support the involvement of distinct 15-LO enzymes between the two cell types.

Our pharmacological data clearly indicate that 15-LO-1 is not responsible for the 15-LO activity we observe in neutrophils. Knowing that 15-LO metabolism in eosinophils is blocked by 15-LO-1 inhibitors, we tested several compounds in order to compare inhibition profiles in eosinophils and neutrophils and found that these profiles are not identical. In eosinophils, we found BLX-3887 and NDGA to be the most potent compounds, as they blocked the synthesis of all measured metabolites in a concentration-dependent fashion. BLX-769 also blocked 15-LO in eosinophils, achieving partial inhibition at 10 μM. Our data in eosinophils also underscore that other documented 15-LO-1 inhibitors (ML351, PD146176, BLX-2481 and BLX-2477) did not have sufficient potency and efficacy to decrease 15-LO metabolism at 10 μM when a fatty acid cocktail was utilized. However, we did test higher concentrations of these inhibitors, and we found that ML351 significantly blocks 15-LO metabolism at 30 μM (data not shown). The fact that the concentrations of inhibitors required to block 15-LO metabolism in eosinophils are consistently higher than those reported in enzymatic assays can be explained by the large amounts of 15-LO metabolites produced by eosinophils (up to 1 nmol/10^6^ cells), in line with a strong expression of the 15-LO-1 enzyme. As for neutrophils, none of the compounds at a concentration of 10 μM significantly inhibited the synthesis of any of the 15-LO metabolites we measured. This allows us to confirm that 15-LO metabolism in neutrophils is independent of 15-LO-1.

Our results also dismiss the possibility of 5-LO being phosphorylated and transformed into a 15-LO in human neutrophils, as was previously suggested [34]. We provide clear evidences that 5-LO phosphorylation does not occur in neutrophils treated with AA and PAF and that 5-LO inhibition, with NDGA or L-739,010, does not affect 15-LO metabolite production while inhibiting that of LTB_4_ (Fig 5). Our data thus eliminates the possibility that phosphor 5-LO or 15-LO-1 are involved and thus, implies that 15-LO metabolism in neutrophils depends on another 15-LO. However, the involvement of the 15-LO-2 in the biosynthesis of 15-LO metabolites by neutrophils is mainly supported by our immunoblot data, which shows that the 15-LO-2 protein is present in neutrophils and absent in eosinophils. In addition, the reported 14-HDHA/17-HDHA production ratio of recombinant 15-LO-2 is ~50 while ours is ~7 [26]. The lack of commercially available selective inhibitors to assess the contribution of 15-LO-2 makes it difficult to absolutely exclude that a third, uncharacterized 15-LO enzyme is implicated in this mechanism.

In conclusion, we demonstrate that human neutrophils can synthesize several 15-LO metabolites independently of 15-LO-1 and very much likely via the 15-LO-2. This biosynthetic pathway of neutrophils might play an important role during host defense, inflammation and/or its resolution, notably via the synthesis of specialized proresolving mediators such as lipoxins, resolvins and protectins [37, 38]. Additional studies using potent and selective inhibitors will certainly refine the involvement of 15-LO-2 in health and disease.

## Supporting information

S1 Dataset This contains the raw data for all figures. (XLSX)
